# Identification of Riboflavin Metabolism Pathway in HepG2 Cells Expressing Genotype IV Swine Hepatitis E Virus ORF3 Protein

**DOI:** 10.3390/vetsci12090912

**Published:** 2025-09-19

**Authors:** Jing Tu, Shengping Wu, Lingjie Wang, Chi Meng, Gengxu Zhou, Jianhua Guo, Jixiang Li, Liting Cao, Zhenhui Song, Hanwei Jiao

**Affiliations:** The College of Veterinary Medicine, Southwest University, Chongqing 402460, China; buffoon@email.swu.edu.cn (J.T.); chemie@email.swu.edu.cn (S.W.); guolicheng666@email.swu.edu.cn (L.W.); mengchi@email.swu.edu.cn (C.M.); zgx973589243@email.swu.edu.cn (G.Z.); guo0619@swu.edu.cn (J.G.); jixianglucky@swu.edu.cn (J.L.); caoliting@swu.edu.cn (L.C.)

**Keywords:** swine hepatitis E virus, HepG2 cells, SHEV ORF3, riboflavin metabolism pathway, circRNA-miRNA network

## Abstract

(1) Background: Hepatitis E (HE) is a novel zoonotic disease caused by hepatitis E virus (HEV). In particular, swine hepatitis E virus (SHEV) genotype IV is one of the main genotypes that infect humans. Open reading frame 3 (ORF3) is an important virulence protein of SHEV, which is involved in virus assembly, release, and regulation of host cell signaling pathways. Circular RNAs (circRNAs), as a type of competitive endogenous RNA (ceRNA), have a closed-loop structure and are special non-coding RNA molecules. They participates in the regulation of multiple biological processes by adsorbing microRNAs (miRNAs). Riboflavin, also known as vitamin B2, is a component of the coenzyme of flavoenzymes in the body. When there is a deficiency of riboflavin, it will affect the biological oxidation process of the host, leading to metabolic disorders. In addition, riboflavin can also affect the synthesis, transportation and decomposition of lipids in the body. It mainly maintains the normal transportation process of fat in the liver. Therefore, the deficiency of riboflavin will lead to the disorder of lipid metabolism in the body. Thus, viral hepatitis is closely related to riboflavin metabolism. However, there are very few reports on SHEV ORF3 affecting the riboflavin metabolism of target cells and thereby influencing viral infection. Therefore, this study investigates this highly significant scientific issue. (2) Methods: In the previous research of our group, adenovirus was used to mediate the overexpression of SHEV ORF3 genotype IV in HepG2 cells. Total RNA was extracted for high-throughput sequencing of circRNAs and transcriptome. KEGG functional enrichment analysis was performed on the data to identify the differentially expressed circRNAs and miRNAs after SHEV infection, and the relevant circRNA-miRNA network in the riboflavin metabolism pathway in HepG2 cells was found. (3) Results: We identified 4 circRNAs in the riboflavin metabolism pathway of HepG2 cells expressing the ORF3 protein of SHEV genotype IV and successfully found 26 relevant circRNA-miRNA networks. (4) Conclusion: We successfully screened and identified circRNAs related to riboflavin metabolism, further identifying the circRNA-miRNA network and its functional targets. For the first time, we investigated the key mechanism by which ORF3 protein influences riboflavin metabolic pathways in target cells through circRNAs, preliminarily revealing that ariboflavinosis can lead to lipid metabolic disorder in the organism. This indicates a close association between viral HE and riboflavin metabolism.

## Simple Summary

Hepatitis E is an infectious disease caused by the hepatitis E virus, with swine hepatitis E virus (SHEV) being capable of infecting humans. This study aims to investigate how a key protein of the virus, ORF3, affects the metabolism of riboflavin (also known as vitamin B2, an essential vitamin that maintains normal fat metabolism in the liver) in human cells through a special type of ribonucleic acid—circular RNAs (circRNAs, a non-coding RNAs with a closed-loop structure). Using gene sequencing technology, we successfully identified four relevant circRNAs and 26 corresponding regulatory network nodes. Our study is the first to reveal the key mechanism by which the ORF3 protein regulates the riboflavin metabolism pathway in host cells through circRNAs and preliminarily confirms that ariboflavinosis can lead to disorders of lipid metabolism in the body. These findings preliminarily demonstrate a significant correlation between viral hepatitis and human riboflavin metabolism, providing new potential targets for the prevention and treatment of the disease.

## 1. Introduction

In recent years, the study of acute severe unexplained hepatitis and viral infection-related liver injury in children has attracted much attention. Research shows that viral infection can exacerbate original liver disease or trigger complex clinical manifestations [1,2]. Among these viral hepatitis-related diseases, hepatitis E virus (HEV), as a significant pathogen, is the fifth known viral hepatitis pathogen in humans and a common cause of acute hepatitis and jaundice worldwide. Similarly capable of inducing acute hepatitis, severe hepatitis, and even extrahepatic manifestations, this virus is particularly prevalent in developing countries, causing substantial economic losses in these regions [3,4,5]. There are four main genotypes of HEV, among which, gene type IV swine HEV (SHEV) is one of the main genotypes of human infections, which is mainly transmitted through the fecal-oral route (such as ingestion of water contaminated with feces or uncooked pork) [6,7,8].

The HEV genome is a single-stranded RNA of 7.2 kb, containing three open reading frames (ORFs). ORF protein is its key virulence-associated protein. It has an ion channel function and can participate in cell signal transduction. It is essential for virus infection and release. Zhan Ding, Brigitte Heller and others have confirmed through experiments that ORF3 protein has a transmembrane structure, can co-locate with endoplasmic reticulum protein, and has non-selective ion channel activity [9,10,11,12].

Riboflavin is the precursor of riboflavin adenine dinucleotide (FAD) and riboflavin mononucleotide (FMN) and participates in redox reactions as a coenzyme to provide energy for cells. In addition, riboflavin can influence the synthesis, transport, and breakdown of lipids in the body, primarily maintaining the normal transport process of fats in the liver [13,14,15]. Studies have shown that riboflavin deficiency can lead to endoplasmic reticulum stress and activation of the unfolded protein response in HepG2 cells, thereby reducing the secretion of apolipoprotein B (apoB) and potentially disrupting lipid balance in the body. Therefore, riboflavin deficiency not only affects the host’s biological oxidation processes, leading to metabolic disorders, but also causes dysregulation of lipid metabolism. Thus, viral hepatitis is closely related to riboflavin metabolism [16,17,18].

Circular RNAs (circRNAs) are a class of non-coding RNAs with a closed-loop structure. It is not affected by RNA exonucleases, and its expression is more stable than other RNAs and is not easily degraded circRNAs are rich in microRNA (miRNA)-binding sites. circRNAs can act as a “molecular sponge” by adsorbing miRNAs to relieve the inhibitory effect of miRNA on target mRNA, thereby increasing the expression level of target genes. Moreover, the circRNA-miRNA network may also regulate the key enzymes and transporters dependent on riboflavin metabolism, thus indirectly regulating the expression of genes related to riboflavin metabolism [18,19,20].

However, up to now, there have been very few reports on the impact of SHEV ORF3 on riboflavin metabolism in target cells. Therefore, this study aims to investigate this highly significant scientific issue. In this study, the overexpression of the genotype IV SHEV ORF3 gene in HepG2 cells mediated by adenovirus was utilized to conduct transcriptome sequencing and perform GO and KEGG functional enrichment analysis. The aim was to explore the relevant circRNA-miRNA network in the riboflavin metabolic pathway in HepG2 cells affected by expressing SHEV ORF3, laying the foundation for further analysis of the SHEV infection mechanism and providing a new perspective on the virus–host interaction. The HepG2 cell line was derived from the liver cancer tissue of a 15-year-old Caucasian person. These cells can secrete various plasma proteins, including albumin, α2-macroglobulin, fibrinogen, and transferrin. Therefore, in HEV research, HepG2 cells are frequently used as model cells [21,22,23]. Hence, this study also employs HepG2 cells as the model cell line.

## 2. Materials and Methods

### 2.1. Overexpression of SHEV ORF3 Genotype IV in HepG2 Cells by Recombinant Adenovirus AD-ORF3 and High-Throughput Sequencing of circRNAs and Transcriptome

HepG2 cells were purchased from the Shanghai Cell Bank of the Chinese Academy of Sciences. After being infected with recombinant adenovirus AD-ORF3 and AD-GFP, respectively, total RNA was extracted, and circular RNA sequencing technology was employed to perform high-throughput sequencing of circular RNAs and transcriptomes [17,18].

In previously published articles, we have successfully achieved overexpression of the ORF3 gene in HepG2 cells. After transfecting HepG2 cells with adenoviruses AD_ORF3 and AD_GFP, green fluorescence signals were observed in all HepG2 cells under an inverted fluorescence microscope, indicating successful expression of enhanced green fluorescent protein (EGFP) and EGFP-ORF3 fusion protein in HepG2 cells. Using GAPDH as an internal reference, T-qPCR was performed to measure the relative expression level of the ORF3 gene. The results showed that the ORF3 gene expression level in the AD-ORF3 experimental group was significantly higher than that in the AD_GFP control group, with a difference of approximately 7800-fold, demonstrating successful adenovirus-mediated overexpression of the ORF3 gene in HepG2 cells. Total proteins were extracted from the AD-ORF3 experimental group and the AD-GFP control group, and Western blot was used to detect ORF3 protein expression levels. The results showed that the β-actin band size in the experimental group was consistent with that of the control group, meeting the total protein quantification standard. A target band appeared at approximately 11.7 kDa in the AD-ORF3 lane of the experimental group, consistent with the expected hypothesis, while no ORF3 expression was detected in the AD-GFP control group, indicating successful overexpression of the type IV SHEV ORF3 gene protein in HepG2 cells. Therefore, in this study, we continued to use HepG2 cells for further research [16,17,18].

### 2.2. Bioinformatics Analysis

Firstly, high-quality total RNA was extracted from Ad_GFP (*n* = 3) and Ad_ORF3 (*n* = 3), a chain-specific library was constructed to remove ribosome RNA (rRNA) from the total RNA. Subsequently, an RNA-seq library was established for high-throughput sequencing. A total of 58,825 genes and 208,460 transcripts were detected through high-throughput transcriptome sequencing, with an average of 3.5 transcripts per gene. Then we used Cutadapt (v21) to carry out quality control of the original sequencing data, and eliminate readings containing joint sequences, low-quality bases and undetermined bases (N). HISAT 2 (v2.2.1) was used to compare the clean reading cutadapt filter with the human genome reference, and Stringtie software (v2.2.3) [24,25] was used to assemble and estimate the expression level of the transcript. The data quality was verified by FastQC software (v0.12.1). To identify circRNAs, quality-controlled reads were aligned to the human reference genome (Homo sapiens, Ensembl release-96) using Bowtie 2 (v2.5.1) and HISAT 2 (v2.2.1). Unmapped reads were subsequently analyzed twice with TopHat-Fusion (v2.0.0) to detect signals potentially generated through back-splicing. CIRCExplorer2 (v2.3.9) and CIRI software (v2.0.6) were employed to predict circRNAs based on structural characteristics and back-splicing junction sequences. The identification criteria were mismatches ≤ 2, back-splicing junction reads ≥ 1, and the distance between the two splicing sites less than 100 kb. Subsequently, the start and end positions of circRNAs were determined, and the results from both software tools were cross-referenced with the CircBase (Date Release) database (which collects and integrates published circRNA data, including information from humans, mice, and other species) to identify known circRNAs (labeled with “hsa-circ” prefixes) and discover novel circRNAs. Differential expression analysis was carried out on the selected cirRNAs, and R-package edgeR was used for differential expression analysis. The criteria for screening differential expression of circRNAs were:|log2(fold change)| ≥ 1 and *p* value < 0.05 [16,17,18].

In our sequencing results, we annotate and enrich the host genes of circRNAs. At present, there is no direct evidence that there is a direct link between circRNAs and the functional annotation of their host genes, the function of circRNAs are reflected through their host genes. Therefore, we performed GO and KEGG enrichment analyses to investigate the host genes of differentially expressed circRNAs. The GO functional enrichment displayed the top 20 pathways (*p* < 0.05), but none were related to the study (Table 1). The KEGG functional cycle enrichment map showed the top 20 pathways (*p* < 0.05) (Table 2). Through the pathway map, the riboflavin metabolism pathway related to other differential pathways was analyzed. Red represents the up-regulation of significantly differentially expressed genes annotated to a certain ko node, blue represents the down-regulation of significantly differentially expressed genes annotated to a certain ko node, and yellow represents that the significantly differentially expressed genes annotated to a certain ko node have both up-regulation and down-regulation. The numbers in the boxes represent the EC numbers of various enzymes, the hollow circles represent small molecular compounds, the solid arrows indicate the direction of biochemical reactions, and the dashed arrows connect other related metabolic pathways.

Based on the gene expression profiles of six samples (AD_GFP1, AD_GFP2, AD_GFP3, AD_ORF3_1, AD_ORF3_2, AD_ORF3_3), the circRNAs with significant differential expression in the riboflavin metabolism pathway were clustered and analyzed, and the expression of circRNAs in different samples was intuitively displayed through a heat map. The horizontal axis in the figure represents the samples, and the vertical axis represents circular RNAs and miRNAs. Different colors represent circRNAs and miRNAs at different levels, and dark blue represents circRNAs and miRNAs with low expression.

### 2.3. Prediction of the circRNA-miRNA Regulatory Network of the Influence of SHEV ORF3 Genotype IV on the Riboflavin Metabolism Pathway in HepG2 Cells

In this study, we mainly performed cis-regulation analysis on circRNAs, and these circRNAs regulate the expression of themselves and adjacent genes. The prediction of the target genes of cis-regulated circRNAs was mainly based on the differential expression of circRNAs and miRNAs within a range of 100 kbp upstream and downstream of the chromosome. Through transcriptome sequencing, 217 differentially expressed genes (a total of 1379 transcripts) were found in HepG2 cells expressing SHEV ORF3 genotype IV, as described previously 18. Therefore, we combined the analysis of circRNAs in the riboflavin metabolism pathway with the transcriptome sequencing results to predict the target genes of cis-regulated circRNAs and explore the circRNA-miRNA regulatory network.

## 3. Results

### 3.1. KEGG Functional Enrichment Analysis: Based on the circRNA Transcriptome Sequencing Data of HepG2 Cells Expressing Genotype IV SHEV ORF3, We Searched for the Riboflavin Metabolic Pathway

**Figure 1:**
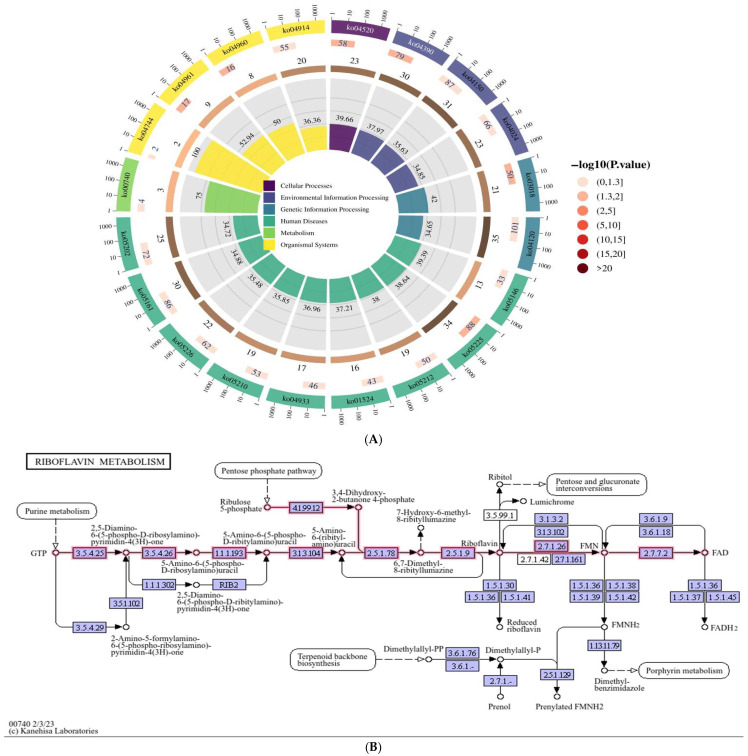
Identification of Riboflavin metabolism (ko00740) in HepG2 cells expressing the ORF3 of genotype IV SHEV. (**A**): The KEGG functional cycle enrichment map shows the top 20 pathways (*p* < 0.05), revealing Riboflavin metabolism (ko00740). (**B**): Schematic diagram of Riboflavin metabolism (ko00740). The sources of Figure 1: KEGG Pathway Database.

The KEGG functional enrichment analysis showed the top 20 pathways (*p* < 0.05), which were: hepatocellular carcinoma (ko05225), RNA degradation (ko03018), Endocrine and other factor-regulated calcium reabsorption (ko04961), Hippo signaling pathway (ko04390), adherens junction (ko04520), Aldosterone-regulated sodium reabsorption (ko04960), mTOR signaling pathway (ko04150), Ubiquitin mediated proteolysis (ko04120), pancreatic cancer (ko05212), riboflavin metabolism (ko00740), Hepatitis B (ko05161), Phototransduction (ko04744), Progesterone-mediated oocyte maturation (ko04914), Amoebiasis (ko05146), Gastric cancer (ko05226), AGE-RAGE signaling pathway in diabetic complications (ko04933), Transcriptional misregulation in cancer (ko05202), Platinum drug resistance (ko01524), Colorectal cancer (ko05210) and cAMP signaling pathway (ko04024) (Figure 1A and Table 2). In existing studies on hepatitis C virus (HCV), it has been found that the HCV life cycle is closely related to host cell lipid metabolism. From viral entry into cells to RNA replication, and further to virion production and assembly, lipids constitute an essential component of the HCV life cycle. As the precursor of FMN and FAD, riboflavin plays a pivotal role in lipid metabolism by influencing lipid synthesis, transport, and degradation in vivo, primarily maintaining normal hepatic fat metabolism [26,27]. Therefore, we selected the riboflavin metabolic pathway (ko00740) in HepG2 cells expressing SHEV ORF3 genotype IV.

The diagram of Riboflavin metabolism (ko00740) is shown in Figure 1B. In the process of riboflavin metabolism in HepG2 cells with the expression of SHEV ORF3 genotype IV, riboflavin is decomposed under the action of multiple enzymes to generate FMD and FAD, which participate in redox enzyme reactions as electron carriers. When a virus invades, it may hijack the FAD synthesis pathway to support its own replication, thereby affecting the invasion of the virus into the body.

### 3.2. Screening of Significantly Differentially Expressed circRNAs in the Riboflavin Metabolism Pathway (ko00740)

**Figure 2:**
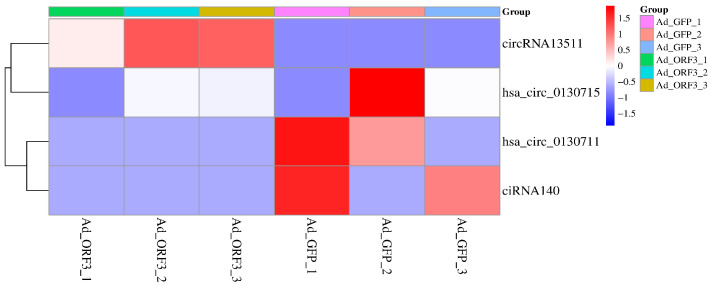
Screening of significantly differentially expressed circRNAs in the riboflavin metabolism pathway (k2o00740) based on circRNA transcriptome sequencing.

In HepG2 cells, we found 2661 significantly different circRNAs (31778 transcripts) and 200 miRNAs, which were mediated by the genotype IV SHEV ORF3, as previously described [16]. In this study, KEGG functional enrichment analysis identified 4 circRNAs in the riboflavin metabolism pathway (ko00740), namely circRNA ENPP3 (circRNA13511), circRNA ENPP1 (hsa_circ_0130715), circRNA ENPP1 (hsa_circ_0130711), and circRNA ENSG00000154269 (ciRNA140), as shown in Figure 2 and Table 3.

### 3.3. Predicting the Potential Target miRNAs of the Four Validated circRNAs and Preliminarily Exploring Their circRNA-miRNA Network

**Figure 3:**
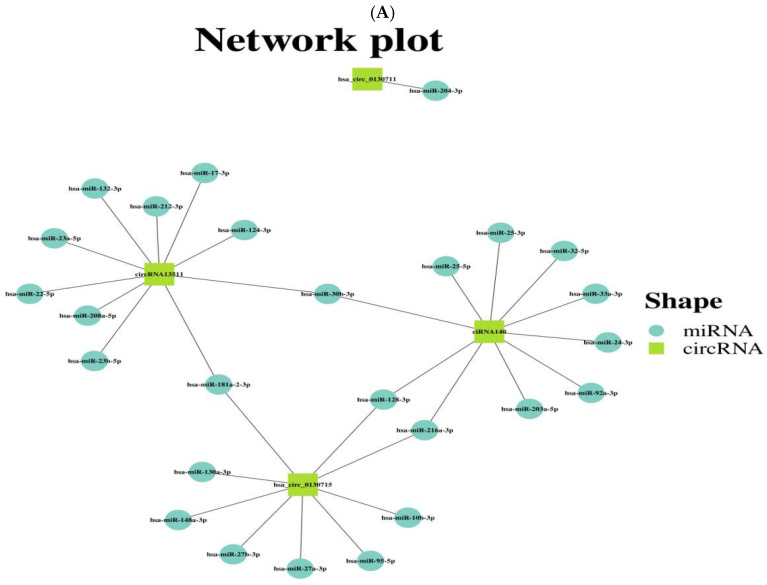
Four circRNAs in the validated riboflavin metabolic pathway (ko00740) had their target miRNAs predicted through the transcriptome. The four validated circRNAs predicted the circRNA-miRNA network, in which the target miRNAs were derived from transcriptome sequencing.

The target genes of circRNA ENPP3 (circRNA13511), circRNA ENPP1 (hsa_circ_0130715), circRNA ENPP1 (hsa_circ_0130711) and circRNA ENSG00000154269 (ciRNA140) were predicted through transcriptome sequencing. It was found that there were 217 differentially expressed genes (1379 transcripts) in HepG2 cells with the expression of genotype IV SHEV ORF3, as previously described [16,17,18]. The results showed that 26 miRNAs were predicted to be the targets of the four validated circRNAs (Figure 3A). Among them, 8 networks are special, namely circRNA ENPP3 (circRNA13511)-has-miR-30b-3p, circRNA ENPP3 (circRNA13511)-has-miR-181a-2-3p, circRNA ENSG00000154269 (ciRNA140)-has-miR-30b-3p, circRNA ENSG00000154269 (ciRNA140)-has-miR-128-3p, circRNA ENSG00000154269 (ciRNA140)-has-miR-216a-3p, circRNA ENPP1 (hsa_circ_0130715)-has-miR-128-3p, circRNA ENPP1 (hsa_circ_0130715)-has-miR-216a-3p, circRNA ENPP1 (hsa_circ_0130715)-has-miR-181a-2-3p (Figure 3A). Among them, has-miR-30b-3p regulates circRNA13511 and ciRNA140, has-miR-181a-2-3p regulates circRNA13511 and hsa_circ_0130715, has-miR-128-3p regulates hsa_circ_0130715 and ciRNA140, and has-miR-216a-3p regulates ciRNA140 and hsa_circ_0130715.

## 4. Discussion

SHE was first discovered by Meng et al. [28], and it is caused by SHEV. In recent years, SHEV has shown an explosive epidemic trend worldwide, and the prevalence of SHEV has been found in more and more developed countries and regions. Therefore, SHE has become a serious public health problem in various countries around the world [29,30,31]. SHEV has four genotypes. Among them, genotype IV is one of the genotypes that mainly infects humans. It first appeared in China in 1993 and is also known as the Chinese genotype. It is the predominantly prevalent genotype in Chinese swine farms at present [32]. The ORF3 protein is a key virulence determinant of SHEV, serving as an indispensable multifunctional protein during viral infection and release, playing a crucial role in its pathogenic process.

Riboflavin, also known as vitamin B2, is a precursor for the synthesis of flavanin coenzyme FAD and FMN, while FAD/FMN is an essential cofactor for hundreds of enzyme reactions. Therefore, the lack of riboflavin can destroy the host’s biooxidation process, lead to metabolic disorders, and widely affect cell metabolism and signal transduction. For example, the lack of riboflavin leads to insufficient FAD and reduced GR activity, which reduces the GSH/GSSG ratio, and finally causes the level of oxidative stress to soar, while high oxidative stress will activate the Keap1-Nrf2-ARE pathway, Nrf2 to initiate the expression of that series of antioxidant genes, and continuous stress will lead to inflammation and cell damage. For example, the lack of riboflavin, FAD and FMD are reduced, and FMD are the cofactors of complexes II and III in the mitochondrial electron transport chain (ETC). Their lack of their lack will directly lead to the decline in ETC efficiency, insufficient ATP generation, the interruption of TCA cycle, causing mitochondrial dysfunction, leading to a large number of active oxygen species. The energy crisis will activate the AMPK signal pathway. Studies have also shown that riboflavin can also affect the synthesis, transportation and decomposition of lipids in the body. It mainly maintains the normal transportation of fat in the liver. Acute riboflavin deficiency can change the proportion of triglycerides and interfere with the beta oxidation of fatty acids, which particularly affects lipid metabolism. In addition, riboflavin deficiency can lead to endoplasmic reticulum stress and activate the protein reaction in HepG2 cells, resulting in fewer lipoprotein B (apoB) secreted by these cells. This may then affect the body’s lipid state. Riboflavin stimulates metabolism and helps digestion and absorption of fat. Therefore, the lack of riboflavin can lead to lipid metabolism disorders. Since the liver is the main organ of lipid metabolism, viral hepatitis is closely related to riboflavin metabolism [33,34,35,36].

Research indicates that ORF3 proteins from multiple hepatotropic viruses collectively influence viral replication and host immunity by interfering with riboflavin (vitamin B2) metabolism. Regarding viral replication, ORF3 disrupts riboflavin metabolism, triggering oxidative stress (increased ROS). This leads to glutathione depletion, thereby stabilizing HBV’s cccDNA and potentially inducing viral polymerase mutations that confer drug resistance. For RNA viruses like HCV, it accelerates their quasispecies evolution. Regarding host physiology, ORF3-mediated FAD deficiency suppresses T-cell function and critical type I interferon (IFN-I) signaling pathways. This weakens the host’s antiviral immune response, conferring an advantage for viral immune escape [37,38,39,40]. Collectively, these studies demonstrate that ORF3 facilitates viral immune evasion by interfering with nuclear flavin metabolism, thereby influencing viral replication. From these studies, it can be inferred that different hepatotropic viruses share commonalities in interfering with riboflavin metabolism. These shared mechanisms may have evolved over long-term viral adaptation to facilitate their survival and transmission within host organisms.

circRNAs are a new type of ncRNAs, and like microRNAs, it is a hot research topic of RNA in recent years [41]. Different from the traditional linear RNA with 5′ and 3′ ends, circRNAs has a closed-loop structure and is not affected by RNA exonucleases. Therefore, circRNAs can be continuously expressed and is less sensitive to degradation. In addition, circRNAs have many miRNA binding sites, so eliminating their inhibitory effects on target genes can enhance their expression. circRNAs can play an important regulatory role in the occurrence and progression of diseases through specific circRNA-miRNA interactions [42,43,44]. Although the regulatory mechanisms of circRNAs in major diseases such as cancer have been extensively studied [45,46,47,48], their roles in viral infections remain largely unexplored. This study is the first to identify key circRNAs (circRNA13511, hsa_circ_0130715, hsa_circ_0130711, and ciRNA140) involved in the riboflavin metabolism pathway (ko00740) mediated by the ORF3 protein of type IV SHEV. While previous studies have demonstrated ORF3’s interference with riboflavin metabolism, no research has yet reported the association between circRNAs and SHEV. Our work reveals for the first time the critical mechanism by which the ORF3 protein influences the riboflavin metabolism pathway in target cells through circRNAs. This also represents the first time the circRNA-miRNA network has been linked to SHEV’s hijacking of riboflavin metabolism, providing novel insights into virus–host interactions.

## 5. Conclusions

In our study, four circRNAs in the riboflavin metabolic pathway (ko00740) were identified in HepG2 cells expressing ORF3 of genotype IV SHEV, namely circRNA ENPP3 (circRNA13511), circRNA ENPP1 (hsa_circ_0130715), circRNA ENPP1 (hsa_circ_0130711), and circRNA ENSG00000154269 (ciRNA140). We predicted 26 circRNA-miRNA networks of them. Four miRNAs regulate two circRNAs, respectively, forming eight networks, which are circRNA ENPP3 (circRNA13511)-has-miR-30b-3p, circRNA ENPP3 (circRNA13511)-has-miR-181a-2-3p, circRNA ENSG00000154269 (ciRNA140)-has-miR-30b-3p, circRNA ENSG00000154269 (ciRNA140)-has-miR-128-3p, circRNA ENSG00000154269 (ciRNA140)-has-miR-216a-3p, circRNA ENPP1 (hsa_circ_0130715)-has-miR-128-3p, circRNA ENPP1 (hsa_circ_0130715)-has-miR-216a-3p, and circRNA ENPP1 (hsa_circ_0130715)-has-miR-181a-2-3p. Our study is the first to investigate the key mechanism by which the ORF3 protein influences the target cell riboflavin metabolic pathway through circRNA, revealing that ariboflavinosis can lead to lipid metabolic disorder in the organism. This also suggests a close association between viral hepatitis E and riboflavin metabolism. In subsequent research, we will focus on evaluating whether these transcriptomic changes translate into functional metabolic alterations and further elucidate the related molecular regulatory mechanisms. This will include overexpression and knockdown experiments, dual-luciferase reporter assays, and Co-IP validation of bioinformatics analysis results to confirm phenotypic changes, as well as quantitative measurement of FMN and FAD concentrations using HPLC or LC-MS/MS methods to assess flavoprotein-dependent enzyme activity and its impact on metabolic disorders.

## Figures and Tables

**Table 1:** The GO functional cycle enrichment map shows the top 20 pathways (*p* < 0.05).

GO_ID	GO_Term	*p*-Value
GO:0031100	animal organ regeneration	0.00
GO:0060412	ventricular septum morphogenesis	0.00
GO:0008420	RNA polymerase II CTD heptapeptide repeat phosphatase activity	0.00
GO:0007406	negative regulation of neuroblast proliferation	0.00
GO:0006886	intracellular protein transport	0.00
GO:0046976	histone methyltransferase activity (H3-K27 specific)	0.00
GO:0098609	cell–cell adhesion	0.00
GO:0070971	endoplasmic reticulum exit site	0.00
GO:0042771	intrinsic apoptotic signaling pathway in response to DNA damage by p53 class mediator	0.00
GO:0003714	transcription corepressor activity	0.00
GO:0046872	metal ion binding	0.00
GO:0047485	protein N-terminus binding	0.00
GO:0005114	type II transforming growth factor beta receptor binding	0.00
GO:0046826	negative regulation of protein export from nucleus	0.00
GO:0001763	morphogenesis of a branching structure	0.00
GO:0008328	ionotropic glutamate receptor complex	0.00
GO:0043175	RNA polymerase core enzyme binding	0.00
GO:0006642	triglyceride mobilization	0.00
GO:0010587	miRNA catabolic process	0.01
GO:0090114	COPII-coated vesicle budding	0.01

**Table 2:** The KEGG functional cycle enrichment map shows the top 20 pathways, revealing Riboflavin metabolism (ko00740) (*p* < 0.05).

Pathway_ID	Pathway_Name
ko05225	Hepatocellular carcinoma
ko03018	RNA degradation
ko04961	Endocrine and other factor-regulated calcium reabsorption
ko04390	Hippo signaling pathway
ko04520	Adherens junction
ko04960	Aldosterone-regulated sodium reabsorption
ko04150	mTOR signaling pathway
ko04120	Ubiquitin mediated proteolysis
ko05212	Pancreatic cancer
ko00740	Riboflavin metabolism
ko05161	Hepatitis B
ko04744	Phototransduction
ko04914	Progesterone-mediated oocyte maturation
ko05146	Amoebiasis
ko05226	Gastric cancer
ko04933	AGE-RAGE signaling pathway in diabetic complications
ko05202	Transcriptional misregulation in cancer
ko01524	Platinum drug resistance
ko05210	Colorectal cancer
ko04024	cAMP signaling pathway

**Table 3:** Test data of 4 circRNAs in the riboflavin metabolism pathway (ko00740).

circRNA Gene ID	circRNA Gene Name	Log2 (Fold Change)	*p*-Value	q-Value	Significant *
circRNA13511	ENPP3	Inf ^a^	0.12	1	yes
ciRNA140	ENSG00000154269	−inf ^a^	0.19	1	yes
hsa_circ_0130711	ENPP1	−inf ^a^	0.26	1	yes
hsa_circ_0130715	ENPP1	−1.21	0.79	1	yes

^a^: An infinite fold change indicates that expression is absent in the control condition. *: When |log2(fold change)| ≥ 1, “significant” is displayed as “yes”.

## Data Availability

The original contributions presented in the study are included in the article, further inquiries can be directed to the corresponding author.
